# Stimulating the Comfort of Textile Electrodes in Wearable Neuromuscular Electrical Stimulation

**DOI:** 10.3390/s150717241

**Published:** 2015-07-16

**Authors:** Hui Zhou, Yi Lu, Wanzhen Chen, Zhen Wu, Haiqing Zou, Ludovic Krundel, Guanglin Li

**Affiliations:** 1 Key Laboratory of Human-Machine Intelligence-Synergy Systems of Chinese Academy of Sciences, Shenzhen 518055, China; E-Mails: hui.zhou@siat.ac.cn (H.Z.); chenwzwx@163.com (W.C.); wzhen8712@126.com (Z.W.); l.a.krundel@lboro.ac.uk (L.K.); 2 Institute of Biomedical and Health Engineering, Shenzhen Institutes of Advanced Technology, Chinese Academy of Sciences, Shenzhen 518055, China; 3 Shenzhen Engineering Lab for Brain Activity Mapping Technologies, Shenzhen Institutes of Advanced Technology, Chinese Academy of Sciences, Shenzhen 518055, China; E-Mail: luyi@siat.ac.cn; 4 Shenzhen Key Laboratory of Electromagnetic Control, Shenzhen University, Shenzhen 518060, China; 5 Nanshan Hospital of Guangdong Medical College, Shenzhen 518052, China; 6 Shenzhen Yingda Strong Technology Co., Ltd, Shenzhen 518112, China; E-Mail: zhaisons@163.com; 7 Electronic Systems Design Laboratory, School of Electronic, Electrical, and Systems Engineering, Loughborough University, Loughborough LE11 3TU, UK

**Keywords:** electrode-electrolyte interface, neuromuscular electrical stimulation, textile electrode, stimulating comfort, finite element modeling

## Abstract

Textile electrodes are becoming an attractive means in the facilitation of surface electrical stimulation. However, the stimulation comfort of textile electrodes and the mechanism behind stimulation discomfort is still unknown. In this study, a textile stimulation electrode was developed using conductive fabrics and then its impedance spectroscopy, stimulation thresholds, and stimulation comfort were quantitatively assessed and compared with those of a wet textile electrode and a hydrogel electrode on healthy subjects. The equivalent circuit models and the finite element models of different types of electrode were built based on the measured impedance data of the electrodes to reveal the possible mechanism of electrical stimulation pain. Our results showed that the wet textile electrode could achieve similar stimulation performance as the hydrogel electrode in motor threshold and stimulation comfort. However, the dry textile electrode was found to have very low pain threshold and induced obvious cutaneous painful sensations during stimulation, in comparison to the wet and hydrogel electrodes. Indeed, the finite element modeling results showed that the activation function along the z direction at the depth of dermis epidermis junction of the dry textile electrode was significantly larger than that of the wet and hydrogel electrodes, thus resulting in stronger activation of pain sensing fibers. Future work will be done to make textile electrodes have similar stimulation performance and comfort as hydrogel electrodes.

## 1. Introduction

Neuromuscular electrical stimulation (NMES) is a commonly used physical therapy method used in human subjects to re-educate motor function [1], increase peripheral blood circulation [2], improve muscle power , and help burning fat [3], *etc.* [4,5]. It would be desirable for users to make NMES therapy devices be convenient and wearable in everyday use for patients with various rehabilitation and healthcare needs. Conventionally, NMES devices mostly use self-adhesive hydrogel electrodes as stimulation electrodes. These gelled electrodes may be advantageous for fixation and good contact with skin surface, but the extended use of hydrogel electrodes can decrease hydrogel resistivity due to drying out, resulting in reduction of stimulation performance [6]. In addition, hydrogel electrodes may cause skin irritation and allergic reactions in patients over time, their repeated use may be unhygienic, and they are not washable or properly sanitized after use. Therefore, these limitations in use of conventional hydrogel electrodes in stimulation devices could make them uncomfortable and inconvenient for long time users. For more comfort and convenient stimulation, textile electrodes fabricated by the integration of conductive yarn into fabrics would be expected to be an alternative to hydrogel electrodes.

In the last decade, more and more interest has been focused on textile electrodes for their potential applications in personal and family health care. Compared to conventional self-adhesive hydrogel electrodes, textile electrodes possess a number of the better features such as being more comfortable to wear and causing almost no skin irritation in long-term use due to their good ventilation, flexibility, foldability, and non-hydrogel characteristics. In addition, textile electrodes can be easily integrated into clothes and conveniently cleaned. With these good properties, textile electrodes should be more suitable in long-term uses such as monitoring physiological signals in clinical applications. Currently, textile electrodes have been used in long-term monitoring of electrocardiogram (ECG) [7,8,9], electromyography (EMG) [10,11,12], electroencephalogram (EEG) [13] signals, and so forth. As an example, self-fabricated textile electrodes have been successfully utilized in our laboratory to record the EMG signals for control of multifunctional myoelectric prostheses [14]. Our results suggested that the textile electrodes could work well in the control of myoelectric prostheses that would be worn by limb amputees for a quite long period every day.

Recently, textile electrodes also have been applied to facilitate electric stimulation in sports and rehabilitation applications [15,16,17,18]. By using various types of washable textile electrodes, Li *et al*. [17] designed an intelligent garment for transcutaneous electrical nerve stimulation. Their experimental results suggested that textile electrodes could achieve similar electrical properties as those of conventional hydrogel electrodes, and could effectively deliver electrical power to the target regions of the human body. The recent study [18] designed a screen printed flexible and breathable fabric electrode array for wearable functional electrical stimulation. The performance tests of the fabric electrode array were conducted on two intact subjects for the involuntary hand and wrist movements by stimulating their wrist and finger extensor muscles. Their experiments showed that the fabric electrode array can produce similar levels of movement as a flexible printed-circuit-board (PCB) array with a hydrogel layer. In addition, in order to improve the stimulation effectiveness and the wearing comfort, different conductive textile materials were investigated and compared in manufacturing textile stimulation electrodes [16]. With integration of conductive textile stimulation electrodes, some smart garments were developed for restoring hand functions [16] and for pain relief [17]. Furthermore, easy and cost-effective fabric manufacturing methods were investigated to make textile stimulation electrode use feasible in both clinical and home environments [18]. These previous studies all suggested that textile electrodes should be an ideal alternative for neuromuscular electrical stimulation in physical therapy and rehabilitation.

Note that it would be expected by users that the conductive stimulation electrodes would provide effective muscle activation at the targeted locations of body with the least pain and without permanent skin damage (burns) and irritation. Although the feasibility and performance of textile electrodes in neuromuscular electrical stimulation have been investigated well in a number of previous studies [15,16,17,18], some important issues such as the impedance spectroscopy, the stimulation comfort, and the underlying discomfort mechanisms of textile electrodes are not well investigated yet. In previous studies [15,19], the stimulation comfort of conventional hydrogel electrodes has been investigated and the results showed that some issues such as the distribution of stimulation current densities, the size and materials of the stimulation electrodes would affect the perceived comfort in neuromuscular electrical stimulation. However, the stimulation comfort and underlying mechanism of textile electrodes in wearable neuromuscular electrical stimulation, which would be significant in their clinical neural rehabilitation applications, remains unknown.

In this study, we used a self-fabricated surface electrode with conductive textiles to investigate its stimulation comfort. The electrode impedance spectroscopies of the dry textile electrode as well as a wet textile electrode and a hydrogel electrode were assessed on healthy subjects. Based on the measured impedance data, the equivalent circuit model was developed and fitting data of different types of electrode were obtained. Moreover, the stimulation threshold and comfort of both dry and wet textile electrodes were analyzed and compared with those of hydrogel electrode in the healthy subjects. Finally, the finite element models of the dry textile electrode, the wet textile electrode and the hydrogel electrode were developed to analyze what causes the stimulation discomfort of the textile electrode. This study might provide some insights into improving stimulation comfort and effectiveness of textile electrodes.

## 2. Methods

### 2.1. Design of a Textile Electrode for Electrical Stimulation

**Figure 1:**
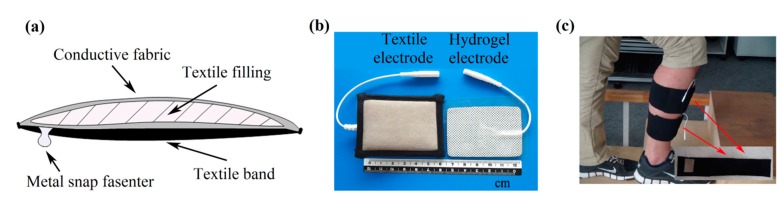
(**a**) A schematic view illustrating the composition of the textile electrode for surface electrical stimulation; (**b**) a photograph of the self-developed textile electrode (left) and a commercial hydrogel electrode (right) used in the experiment; (**c**) a photograph of the placement of the developed textile electrode on a subject.

The studied textile electrode consisted of conductive fabric, textile filling, textile band, and metal snap fastener, as shown in Figure 1. The conductive fabric was made of silvered polyamide with additional Spandex. Absorbent sponge was used as textile filling, which was enwrapped with the conductive fabric. The textile band was used as a textile base. The metal snap fastener was employed for connection with stimulation devices. An elastic Velcro strap was utilized to locate the textile electrode at the proper location on the legs in the functional electrical stimulation application. The dimension of each layer of the textile electrode is about 6 cm × 4 cm, and the thicknesses of the conductive fabric and the absorbent sponge are about 0.4 mm and 5 mm, respectively.

### 2.2. Impedance Spectroscopy Measurement of the Textile Electrode

In order to estimate the impedance spectroscopy of the self-fabricated textile electrode, ten healthy subjects (four females and six males, aged 28.4 ± 2.59 years) participated in the electrode-skin impedance spectroscopy measurement experiments. The recruitment process of subjects and the experimental protocols were approved by the Ethics Committee for Human Research, Shenzhen Institutes of Advanced Technology, Chinese Academy of Sciences. All subjects were informed about the purpose and the procedures to be used in the study. The electrode-skin impedance was measured using a Gamry Potentiostat (Reference 600) with a 10-mV (RMS) AC sinusoid signal in a frequency range of 0.1 Hz to 100 kHz. A two-electrode cell configuration was setup in the Gamry system.

**Figure 2:**
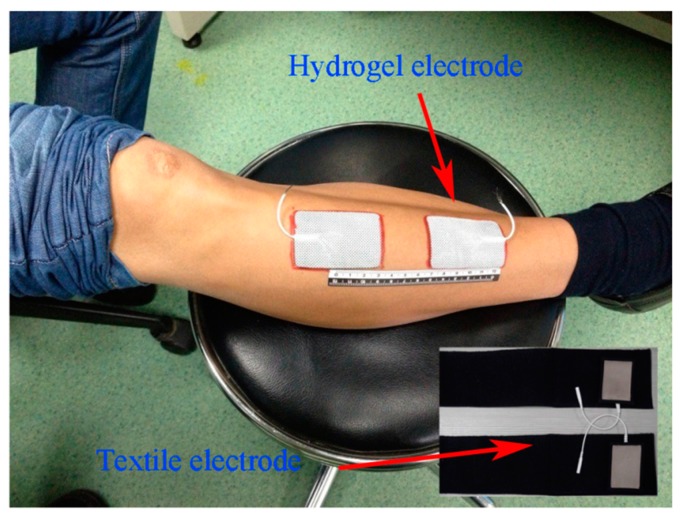
Placement of electrodes for impedance spectroscopy measurement.

The subjects were seated on a chair with the tested lower leg horizontally placed on a table with adjustable height. The skin area over the tibialis anterior (TA) muscle was cleaned with 75% isopropyl alcohol before the placement of each type of electrode. A pair of electrodes (either textile or hydrogel) were placed over the TA muscle with a proximal inter-electrode distance of 4 cm, as shown in Figure 2. For each subject, two types of textile electrodes (dry one and wet one) were used, respectively. A wet textile electrode was made by pouring about 2 mL of pure water onto a dry textile electrode.

**Figure 3:**
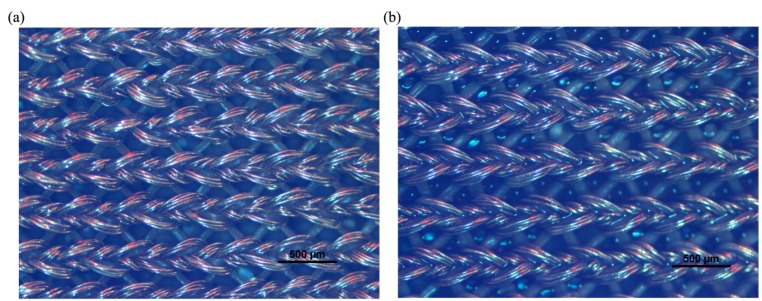
Microscopic view of two textile electrodes: (**a**) the dry textile electrode; (**b**) the wet textile electrode.

Microscopic views of the dry and wet textile electrodes are shown in Figure 3, from which we could see that there are some water drops between the textile grids in the wet electrode (Figure 3b). For comparison purposes, a commercially available common hydrogel electrode (Nanjing Dalun Medical Technology Co. Ltd., Nanjing, China) was also used in the experiments. All these electrodes had a similar size of approximately 6 cm × 4 cm. For each subject, the three types of electrode would be mounted on his/her TA muscle in a random order, respectively. The electrode locations were marked with a pen to ensure that the replacement of each type of electrodes could be at the same sites.

### 2.3. Stimulation Thresholds and Comfort Evaluation

To estimate the stimulation thresholds and comfort, we recruited ten healthy subjects (six males and four females, aged 23.4 ± 1.8 years) for the experiments of electrical stimulation of the TA muscle. The protocols of the stimulation experiments were approved by the Ethics Committee for Human Research, Shenzhen Institutes of Advanced Technology, Chinese Academy of Sciences. All the subjects were informed about the purpose and the procedures of the study. The subjects were seated on a chair with their foot not touching the floor. The skin area over the TA muscle was cleaned with 75% isopropyl alcohol before the placement of each type of electrodes. An evoked potential system (NCC Medical, Shanghai, China) was used to produce monophasic rectangular stimulation pulses that were used in the experiment to estimate the sensory, pain, and motor thresholds of the three different electrode types. The stimulation pulse was set at a frequency of 30 Hz with a 200 μs pulse width. The stimulation current amplitude was gradually increased from the initial value of 1 mA with 1 mA steps to find the sensory, motor, and pain thresholds, respectively, in four consecutive stimulation sessions, as described previously in [19]. The first two sessions allowed subjects to become accustomed to the surface stimulation sensation, and then another two were used to calculate the mean values of the thresholds of the three sensing levels to electrical stimulation.

The sensory threshold to electrical stimulation was the amplitude of stimulating current at which the subjects started feeling the electrical stimulation on their TA muscle. The motor threshold was recorded as the stimulation amplitude when the visible foot dorsiflexion movement was first observed with increasing stimulation current strength. When the subjects felt obvious pain along with increasing stimulation current strength, the amplitude of the stimulating current was considered as the pain threshold. Note that if the pain threshold of a subject was reached before inducing a visible foot dorsiflexion movement, his/her motor threshold would be unavailable. For every subject, three types of electrodes (dry and wet textile as well as hydrogel) were tested in a random order for determining the stimulation thresholds of the three sensing levels, respectively.

For each of the subjects, the stimulation comfort was evaluated at the stimulation amplitudes corresponding to his/her pain thresholds (Pth) with three different electrodes, respectively. The subjects would be asked to rate stimulation sensations according to the transcutaneous electrical stimulation comfort questionnaire (TESCQ) scale developed by Lawrence [19]. In brief, TESCQ is a modified form of the short term McGill pain questionnaire, with 14 different sensations related to cutaneous, deep and general sensory receptors. In the experiments of this study, the hydrogel, wet textile, and dry textile electrodes were applied in a randomized order at the same stimulation sites on the TA muscle. The total score for cutaneous, deep and general categories was calculated and displayed to compare stimulation comfort of different types of electrodes.

### 2.4. Modeling of Transcutaneous Electrical Stimulation

**Figure 4:**
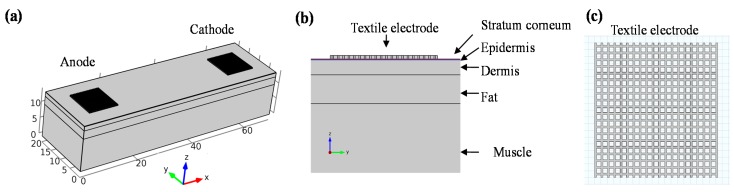
Finite element model of dry textile electrode; three dimensional view of the model is shown in (**a**), side view is shown in (**b**), and the details of modeled dry textile electrode is shown in (**c**).

In order to more deeply understand discomfort sensations during stimulation, three dimensional finite element models of different electrode types were developed with the aid of software (COMSOL Multiphysics 4.3a, COMSOL, Stockholm, Sweden). The skin model represented a 2 cm × 7 cm area of human skin, which consisted of five horizontal layers: stratum corneum, epidermis, dermis, fat, and muscle, as illustrated in Figure 4. The thickness and electrical parameters of each layer are listed in Table 1. For the dry textile electrode, since the conductive fabric touched with skin directly, only a layer of square shaped sheet with a thickness of 0.4 mm was modeled (see Figure 4). The conductivity value of dry textile electrode was estimated from the measured resistance of conductive fabric. The line width was set at 0.15 mm with a separation of 0.35 mm by approximate estimation from the microphotos of dry textile electrodes. Besides, the wet textile electrode could be treated as homogeneous conductive solution similar to 0.9% NaCl. Then the conductivity of wet textile electrode was set as 1.4 S/m in the model. The thickness of the wet textile electrode was set as 5.8 mm, which was estimated from two layers of conductive fabric (0.4 mm each) and a sandwiched absorbent sponge (5 mm). The hydrogel electrode was modeled as a foil and a layer of hydrogel [20]. All electrodes were modeled as 1 cm length × 1 cm width. A stationary electrical field of the skin area was developed in the model [21]. Both the anode (return) and cathode electrodes were placed on the surface of stratum corneum with a separation distance of 6 cm. The top surface of the anode electrode was set as the ground and the top surface of the cathode electrode was set as the stimulation current source (−2 mA).

**Table 1:** The electrical conductivity and thickness of each layer in the finite element model.

Layer	Electrical Conductivity (S/m)	Thickness (μm)
Stratum corneum	2 × 10^−5^ [22,23]	29 [24,25]
Epidermis	Horizontal:0.95, vertical:0.15 [20,22]	60 [24,25]
Dermis	Horizontal:2.57, vertical:1.62 [20,22]	1300 [26,27]
Fat	0.04 [28]	2500 [29]
Muscle	Horizontal:0.25, vertical:0.75 [28]	10000 [29]
Textile electrode sheet	1.4 × 10^5^	Thickness: 400, line width:150
Wet textile electrode	1.4 [30]	5800
Hydrogel	4.6 × 10^−3^	1000
Foil electrode	6.67 × 10^5^ [20]	50

During transcutaneous electrical stimulation, discomfort feeling is mainly related to the activation of Aδ fiber. The pain sensing Aδ fibers usually terminate in the junction of epidermis and dermis [21]. Thus it can be assumed as a vertical direction from epidermis towards fat for simplicity. Therefore, the electric field gradient (activation function) along the vertical direction can be used to describe the activation of Aδ fibers in response to transcutaneous electrical stimulation with different electrode types.

## 3. Results

### 3.1. Electrode Impedance Spectroscopy

**Figure 5:**
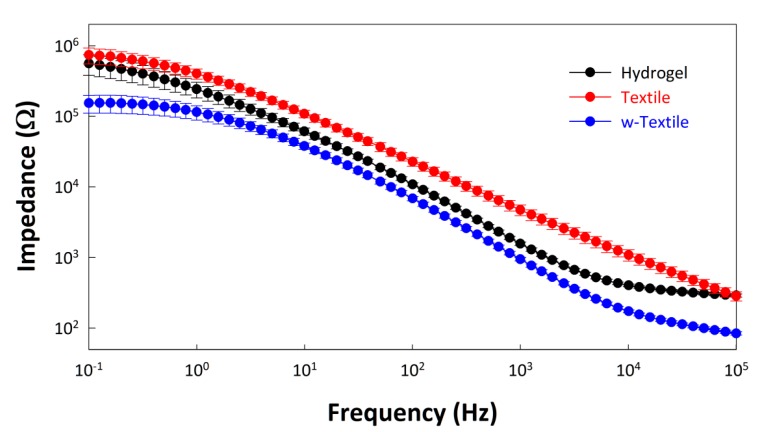
Impedance of the hydrogel electrode, textile electrode, and wet textile electrode (w-textile electrode) tested on TA muscle of human legs.

As a steady-state technique, electrochemical impedance spectroscopy (EIS) is a powerful tool for the assessment of electrode interfaces. The average EIS results of the hydrogel electrode, textile electrode, and wet textile electrode (w-textile electrode) measured on the subjects are demonstrated in Figure 5 with a logarithmic plot. It can be seen from Figure 5 that the impedance of the textile electrode was higher than that of the hydrogel electrode, but after fully absorbing the water, the impedance of the wet-textile electrode decreased significantly across the whole frequency interval. The average impedance at 1 kHz reduced from 4.72 kΩ of the dry textile electrode to 0.95 kΩ of the wet textile electrode, about 40% lower than the hydrogel electrode (1.57 kΩ).

**Figure 6:**
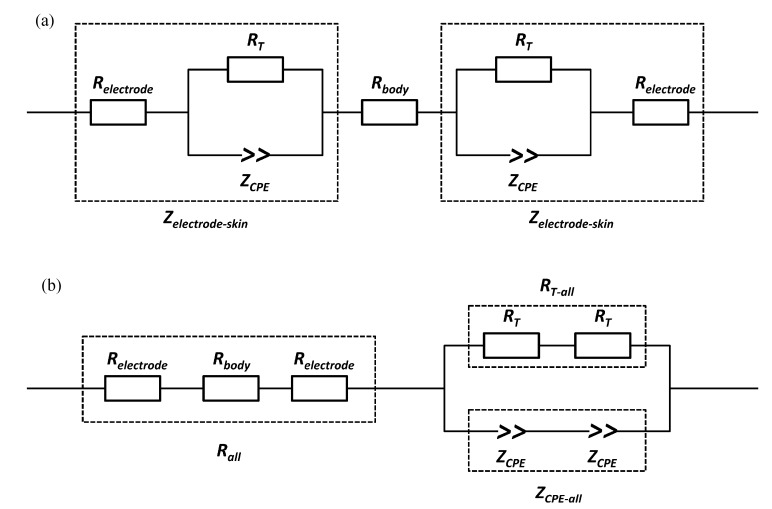
(**a**) Developed equivalent circuit model for the electrodes tested on leg muscle, with circuit elements including the electrode-skin impedance (Z_electrode-skin_) and the body resistance (R_body_). The Z_electrode-skin_ is composed by electrode resistance (R_electode_), and charge-transfer resistance (R_T_) and double-layer constant-phase element (Z_CPE_) at the electrode-skin interface; (**b**) A simplified equivalent circuit model for data fitting, with circuit elements including the total resistance (R_all_) of body (R_body_) and electrode (R_electode_), total charge-transfer resistance (R_T-all_) and total double-layer constant-phase element (Z_CPE-all_) at the electrode-skin interface.

In order to facilitate data fitting, a simplified equivalent circuit model was proposed (shown in Figure 6), where R_all_ is the total resistance of the body (R_body_) and electrode (R_electrode_), R_T-all_ and Z_CPE-all_ represent the total charge transfer resistance (R_T_) and double-layer constant-phase element (Z_CPE_) at the electrode-skin interface, respectively. The constant phase element (CPE) was introduced to represent the dissipative double-layer capacitance, which reflects the characteristics of a microscopic fractal at blocking electrode-electrolyte interfaces. The concept of CPE could be explained with the following equation:

Z*_CPE_* = 1/[q(*j*ω) ^n^] (1)

where *j* = √-1, ω is the angular frequency (rad∙s^−1^) = 2π*f*, and *f* is frequency in Hz. The parameters of the CPE are defined by q and n. The parameter q indicates the value of the capacitance of the CPE as n approaches 1, and it has the numerical value of 1/Z_CPE_ at ω = 1 rad/s. The parameter n reveals the micro fractal and distribution of the phase-phase interface. It correlates to energy dispersion on the electrode-electrolyte interface and can be affected by a series of factors, such as surface roughness, distribution of reaction rates, or a non-uniform current distribution. When n = 1, this CPE is identical to a capacitor.

**Figure 7:**
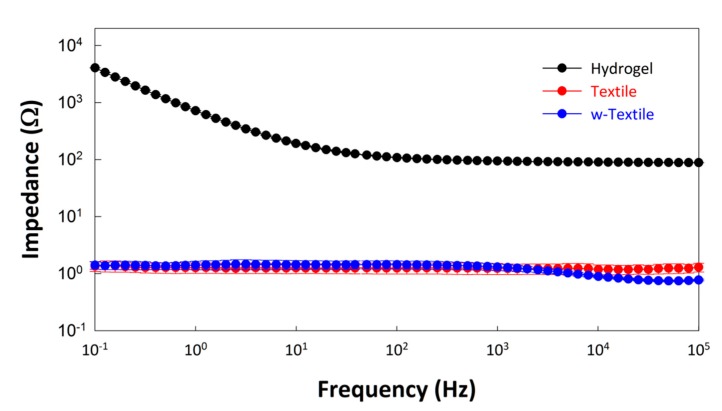
Impedance of the hydrogel electrode, textile electrode, and wet textile electrode tested on large-area stainless steel plate.

To determine the electrode resistance, all three types of electrodes (hydrogel electrode, textile electrode and w-textile electrode) were also placed one by one on a stainless steel plate with a length of 8 cm and a width of 6 cm. A bandage was used to maintain good contact between the electrode and the stainless steel plate. The AC impedance of the electrodes on the stainless steel plate was measured in the frequency range between 0.1 Hz and 100 kHz and presented in Figure 7. It can be seen from Figure 7 that the impedances of all the three types of electrodes at the whole testing frequency range were almost independent of frequency. The value of R_electrode_ was obtained from data fitting using the equivalent circuit in Figure 6b.

The fitting data for the proposed equivalent circuit model is summarized in Table 2. As Table 2 indicates, the electrode resistance of the hydrogel electrode was significantly higher than those of the textile and w-textile electrodes. This suggests that the hydrogel component of the electrode may hinder the transfer of ions within the polymer film and subsequently increase the charge transfer resistance. The dry textile electrode shows the highest charge transfer resistance and lowest CPE-n value, which is presumably due to the poor contact between the textile and skin. However, after being fully absorbed in water, the charge transfer resistance dramatically reduced and the capacitance (CPE-q) increased from 1.04 μF^n-1^ to 1.52 μF^n-1^, 50% higher than the hydrogel electrode. Besides this, the CPE-n value increased to 0.83, which is approximated to an ideal capacitor.

**Table 2:** Fitting data for proposed equivalent circuit model shown in Figure 6b. Statistical results were expressed as mean ± S.E.M.

Parameters	Unit	Hydrogel	Textile	w-Textile
*Z (at 1 kHz)*	Ω	1572.3 ± 83.78	4716.7 ± 815.60	947.5 ± 53.65
*R_all_*	Ω	269.2 ± 9.13	97.1 ± 21.5	80.18 ± 4.73
*R_T-all_*	kΩ	304.1 ± 109.79	477.0 ± 141.69	79.3 ± 24.17
CPE-q	μF^n-1^	0.98 ± 0.11	1.04 ± 0.12	1.52 ± 0.14
CPE-n	0 ≤ n ≤ 1	0.83 ± 0.01	0.70 ± 0.02	0.83 ± 0.01
*R_electrode_*	Ω	90.98 ± 1.44	1.33 ± 0.35	0.80 ± 0.02
*R_body_*	Ω	87.24	94.44	78.58

### 3.2. Stimulation Thresholds

The mean stimulation thresholds of the three types of sensation (sensory, motor, and pain) for different electrodes are listed in Table 3. A paired t-test was used in the statistical computation. There were significant differences in sensory thresholds between hydrogel electrode (6.3 ± 0.59 mA) and dry textile electrode (2.45 ± 0.34 mA), and between the wet textile electrode (5.9 ± 0.65 mA) and dry textile electrode. However, there was no significant sensory threshold difference between the hydrogel electrode and wet textile electrode. The results also showed a significant difference in motor thresholds between hydrogel electrode (19.9 ± 1.29 mA) and wet textile electrode (21.6 ± 1.42 mA). Note that the motor threshold for the dry textile electrode was not be available (Table 3) because the subjects felt obvious pain before the stimulation current induced a visible foot dorsiflexion movement, so the electrical stimulation experiment was stopped to avoid causing intolerable pain to the subjects. With regard to pain thresholds, dry textile electrode showed the lowest threshold (4 ± 0.47 mA), while no significant differences were found between hydrogel electrode (33.15 ± 2.01 mA) and wet textile electrode (31.90 ± 2.01 mA).

**Table 3:** Comparison of the mean stimulation thresholds of sensory, motor and pain for different types of electrode. Statistical results are expressed as mean ± S.E.M. NA denotes not available.

Threshold (mA)	Hydrogel	Wet Textile	Dry Textile
Sensory	6.3 ± 0.59 *^1^	5.9 ± 0.65 *^2^	2.35 ± 0.30
Motor	19.9 ± 1.29 ^†^	21.6 ± 1.42	NA
Pain	33.15 ± 2.01 ^‡1^	31.90 ± 2.01 ^‡2^	3.2 ± 0.53

Superscripts indicate a significant difference in sensory threshold between: *^1^ the hydrogel and dry textile electrodes, *p* < 0.05; *^2^ the wet textile and dry textile electrodes, p < 0.05; ^†^ the hydrogel and wet textile electrodes, *p* < 0.05; or a significant difference in pain threshold between: ^‡1^ the hydrogel and dry textile electrode, *p* < 0.05; ^‡2^ the wet textile and dry textile electrodes, *p* < 0.05.

### 3.3. Comfort Evaluation

**Figure 8:**
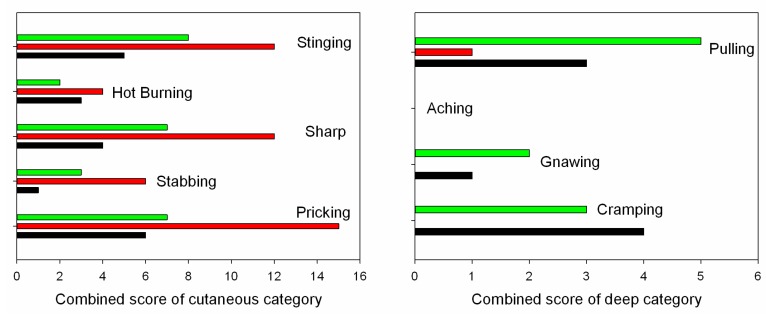

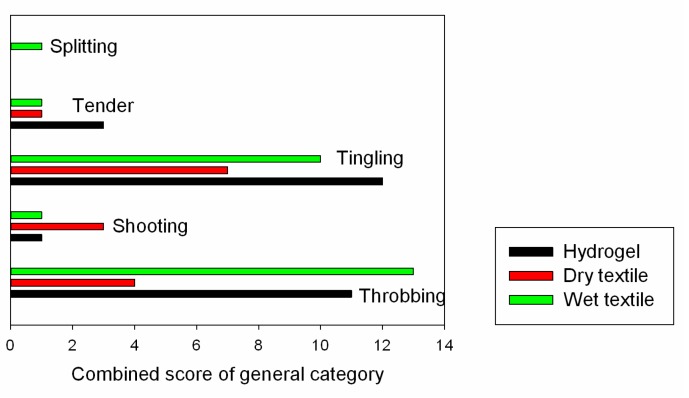
The sums of TESCQ scores of cutaneous, deep and general categories with the three different electrode types from all the subjects.

Subjects were asked to rate the stimulation comfort of the different electrode types using the TESCQ questionnaire at 1хPth stimulation intensity. The sums of TESCQ scores from all the subjects are shown in Figure 8. At the 1хPth stimulation intensity, the cutaneous related stinging, hot burning, sharp, stabbing, and pricking pain sensations were distinct for different types of stimulation electrodes. With respect to the deep categorized pulling, aching, gnawing, and cramping pain, dry textile electrode only showed a score for pulling, while the wet textile electrode and hydrogel electrode showed moderate scores of pulling, gnawing and cramping. With regard to the general category, tingling and throbbing were the prominent sensations for all electrode types. The sums of scores of cutaneous, deep and general category for each electrode type from all the subjects are summarized in Table 4. While the total cutaneous score of the dry textile electrode was significantly higher than those of the hydrogel electrode and wet textile electrode, the total deep and general scores of dry textile electrode were much less than those of the other electrode types. Furthermore, the total cutaneous score of the wet textile electrode was slightly higher than that of the hydrogel electrode, but the deep and general scores of the wet textile electrode were similar to that of the hydrogel electrode.

**Table 4:** Sum of TESCQ scores for different types of electrode from all the subjects.

Electrode Type	Cutaneous Pain	Deep Pain	General Pain
Hydrogel	19	8	27
Dry textile	49	1	15
Wet textile	27	10	26

### 3.4. Finite Element Analysis

**Figure 9:**
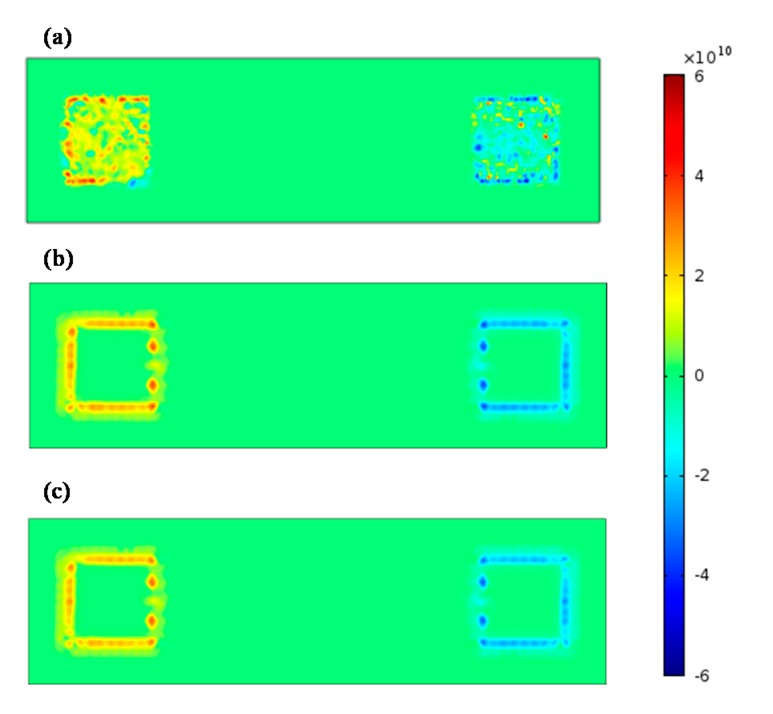
The electric field gradient at the depth of epidermis-dermis junction for dry textile electrode (**a**), wet textile electrode (**b**), and hydrogel electrode (**c**). In the figure, the anode electrode is located on the left side while the cathode electrode is located on the right side.

Since pain receptors are normally located in the dermis, so the electric field gradient along the z direction was critical in the determination of painful feelings. The electric field gradient at the depth of the epidermis-dermis junction was simulated and plotted in Figure 9 for the comparison of pain sensing fiber activation when using the different electrode types.

In Figure 9, the hot spots (high values of activation function) were mainly located below the anode electrode of all the electrode types. Besides, hot spots appeared below the silvered threads of the dry textile electrode, while the hot spots only appeared around the edges of the hydrogel and wet textile electrodes. Compared with other electrodes, the dry textile electrode showed strong activation function along the z direction (normal direction).

## 4. Discussion

Textile electrodes have been developed as a new type of surface electrical stimulation electrode that may replace traditional hydrogel electrodes in wearable healthcare applications due to their excellent long term use, washability, convenience, breathability, and easy integration in clothes. Previous studies have shown that as electrical stimulation electrodes textile electrodes could display similar electrical properties as conventional hydrogel electrodes [17,18]. However, the stimulation comfort of textile electrodes needs to be assessed, which would be an important feature for their practical use. In this study, the stimulation characteristics and comfort of a textile electrode were investigated and compared with those of a hydrogel electrode and a wet textile electrode. Our results demonstrated that a wet textile electrode can have similar stimulation thresholds of sensations and comfort as a hydrogel electrode. Using a wetting textile electrode could improve the contact characteristics of the electrode-skin interface for better stimulation comfort in practical applications. For example, a wetted fabric electrode has been used by Bioness Inc. (Santa Clarita, CA, USA) [31] to improve the stimulation performance. It is true that maintaining the moisture of a textile electrode for a long time would be difficult, but it is still feasible. For example, in the work of Weder [32], the textile electrode was integrated with a small water reservoir to guarantee wetting of the textile for a long time. Besides, Keller has suggested that an additional interface layer such as hydrogel or some new skin interface material would be needed in order to improve the contact between the textile electrode and the skin [15]. Many endeavors have now been put into the area of textile electrodes to overcome the challenges of the electrode skin interface and improve the stimulation comfort of this new type of electrode.

Electrical impedance characteristics affect the stimulation efficiency and selectivity. It was reported that the stimulation efficiency and focality were lower in low resistivity hydrogel electrodes compared to high resistivity hydrogel ones [6]. However, only simulation results were presented in that study. In our study, with the same application of TA muscle stimulation for dorsiflexion, the motor threshold of a low resistive electrode (wet textile electrode) was statistically lower than that of a high resistive electrode (hydrogel electrode) in experiments on healthy subjects. This illustrates how electrode impedance influences the activation of motor neuron fibers. In transcutaneous electrical stimulation applications, it is important to design proper electrode impedance parameters to activate the targeted neural fibers.

An interesting finding of the finite element modeling was that the activation zone is mainly located below the anode electrode. In previous models [6,28,33], activated nerves were parallel to the anode cathode axis and located at muscle level depth. The cathode electrode produces a positive activation function value while the anode electrode produces negative activation function value, which indicates that the activated zone is located under the cathode electrode. In our model, the activation function along the z direction at the depth of epidermis-dermis junction was used to explain painful sensations during surface electrical stimulation. The modeling results showed a strong activation under the textile electrode anode. Furthermore, in our electrical stimulation experiments on subjects, they did experience a strong painful feeling under the anode electrode using the dry textile electrode at low stimulus intensities. It is widely accepted that an “edge biting” effect would be experienced by subjects using hydrogel electrodes. Our simulated results from the FEM model also showed a strong activation of pain sensing fibers at the edge of the hydrogel electrode, as shown in Figure 9, which is in accord with this “edge biting” effect. These simulated results suggest that by using the FEM model some reasonable explanations of a possible mechanism of the painful feelings caused by surface electrical stimulation of a textile electrode could be proposed. Note that like previous relevant model studies [20,28,33], the FEM model used in this study is also simple. It may be necessary to use a more elaborate FEM model based on the anatomical structure of human body as well as electrophysiology to reveal the mechanism of electrical stimulation pain.

Although the developed model can explain the painful feelings of dry textile electrodes at low current levels, the model still has some limitations. A simplified layered geometry was used to represent the human skin without considering the influence of pores and glands. The tissue of each layer was assumed as purely resistive, and the capacitive and dispersive properties of tissue were neglected. Furthermore, the electrode-skin interface characteristics such as capacitive or pseudo-capacitive coupling were not included in the model. The activating function at the specific depth along the z direction was used to describe the pain sensing fiber activation situation in the model. In fact, the Aδ fiber may have a complex geometric shape and orientation [21,34].

## 5. Conclusions

This study has investigated the electrical stimulation comfort of textile electrodes in comparison with traditional hydrogel electrodes. The electrical impedance spectral analysis showed a poor contact between the dry textile electrode and skin, while this contact was improved with a wet textile electrode. The stimulation experiment demonstrated that dry textile electrodes may cause pain at very low electric currents, while the wet textile electrode and hydrogel electrode can activate foot dorsiflexion movements without pain. The reason behind this is indicated by modeling results that the pain sensing fiber will be activated more readily by a dry textile electrode than a wet textile electrode or hydrogel electrode. Further work needs to be conducted in order to improve the electrode skin contact of dry textile electrodes.
